# Postoperative fluid balance and outcomes after Pancreaticoduodenectomy: a retrospective study in 301 patients

**DOI:** 10.1007/s00423-022-02443-6

**Published:** 2022-02-22

**Authors:** Hang Zhang, Yechen Feng, Duoji Suolang, Chao Dang, Renyi Qin

**Affiliations:** Department of Biliary-Pancreatic Surgery, Affiliated Tongji Hospital, Tongji Medical College, Huazhong University of Science and Technology, 1095 Jiefang Avenue, Wuhan, 430030 Hubei China

**Keywords:** Fluid management, Pancreaticoduodenectomy, Whipple, Outcomes

## Abstract

**Background:**

The incidence of postoperative morbidity after pancreaticoduodenectomy (PD) is high; however, whether fluid management after surgery affects postoperative morbidity is unclear. This study aimed to determine whether fluid balance in patients undergoing PD is associated with postoperative complications and mortality.

**Methods:**

Data from a computer-based database of patients who underwent PD between 2016 and 2019 were retrospectively analyzed. Patients were stratified into four quartiles according to their fluid balance at 0–24, 24–48, 48–72, and 72–96 h after surgery. The predefined primary outcome measures were morbidity and mortality rates.

**Results:**

A total of 301 patients were included. The morbidity and mortality rates in the cohort were 56.5% and 3.7%, respectively. The most common complications after PD were postoperative pancreatic fistula (31.9%) and delayed gastric emptying (31.6%). Patients with a higher fluid balance in the 0–24-, 24–48-, and 48–72-h postoperative periods had a higher morbidity rate and longer hospital stay than those with a lower fluid balance (all *P* < 0.05). Patients with a fluid balance of 4212 mL during the postoperative 0–72 h were most likely to develop complications (*P* < 0.001). The area under the receiver operating characteristic curve was 0.71 (0.65–0.77), with a sensitivity of 58.24% and a specificity of 77.10%.

**Conclusions:**

Higher postoperative fluid balance seems to be associated with increased morbidity after PD compared to lower fluid balance. Surgeons should pay close attention to the occurrence of complications in patients with a high fluid balance.

**Supplementary Information:**

The online version contains supplementary material available at 10.1007/s00423-022-02443-6.

## Introduction

Pancreaticoduodenectomy (PD) remains one of the most challenging surgical procedures and has the highest complication rate among all abdominal surgical procedures. Although the perioperative mortality rate associated with PD has decreased in recent decades from >20% to <3%, especially in high-volume centers [1], the morbidity rate remains high, ranging from 40% to 60% [2]. Considering the high complication rate, one of the principal goals of clinical research related to PD during the past few decades has been to reduce postoperative morbidity. The methods proposed to reduce morbidity after PD include the use of octreotide and its analogs or other pharmacologic agents, modifications in the type of surgical process, and variations in anastomotic methods [3–6]. Some of these techniques have improved the outcomes of PD, although others remain unsatisfactory. One largely unexplored strategy that has been proposed to reduce complications and improve outcomes after PD is control of perioperative fluid administration.

Perioperative fluid management can be challenging. The effects of third spacing and evaporative losses in decreasing extracellular fluid volume have led to the use of traditional aggressive intravenous fluid support, especially during complicated operations such as PD. [7] However, only a few studies have examined the impact of postoperative fluid balance on outcomes in this high-risk population of patients undergoing PD. Therefore, this retrospective study aimed to evaluate whether perioperative fluid administration is associated with the short-term outcomes after PD.

## Methods

Patients who underwent PD between April 2016 and February 2019 were retrospectively analyzed. The inclusion criterion was undergoing the PD procedure. Patients who underwent other surgical procedures because of metastasis or changes in the surgical plan (central pancreatectomy, distal pancreatectomy, or gastrojejunostomy) were excluded. A computer-based database was searched for data including age, sex, surgical procedure, pathologic characteristics, length of hospital stay, morbidity, and mortality. The body surface area was calculated using height and weight measurements. All surgical procedures were performed by a single experienced surgeon (RYQ). Fluid intake and output data were collected at 24, 48, 72, and 96 h postoperatively. Fluid balance was defined as the intake volume minus the output volume at each time interval. For analysis, patients were divided into quartiles according to the overall fluid balance during each time interval (0–24, 24–48, 48–72, and 72–96 h postoperatively). The predefined primary outcome measures were morbidity and mortality rates. Morbidities were graded using the Clavien–Dindo classification system [8]. Secondary outcome measures included hospital length of stay (LOS), intensive care unit (ICU) LOS, surgical-site or abdominal infection, delayed gastric emptying (DGE), postoperative pancreatic fistula (POPF), bile leakage, bowel leakage, acute kidney injury (AKI), major cardiopulmonary complications, and hospital readmission within 90 days after surgery. POPF was defined in accordance with the guidelines of the International Study Group of Pancreatic Fistula [9]. DGE was defined in accordance with the guidelines of the International Study Group of Pancreatic Surgery [10]. AKI was defined as a 50% increase in serum creatinine level from baseline. Systemic inflammatory response syndrome (SIRS) was defined in accordance with the definition of the American College of Chest Physicians/Society of Critical Care Medicine Consensus Conference [11]. Acute respiratory distress syndrome (ARDS) was defined in accordance with the Berlin definition [12]. The study was approved by the ethics committee of Tongji Hospital.

The summary statistics of the study population were tabulated. The area under the receiver operating characteristic curve of fluid input was calculated. The cutoff value of fluid input for identifying morbidity was obtained and evaluated for sensitivity and specificity. Statistical significance was set at *P* < 0.05. Statistical analyses were performed using IBM SPSS (version 22.0; IBM, Armonk, NY, USA) and SAS (version 9.4; SAS Institute, Cary, NC, USA).

## Results

A total of 304 patients underwent PD during the study period. Three patients with missing data were excluded from this study. The demographic data of the remaining 301 patients are presented in Table 1. Of the patients, 97.06% had American Society of Anesthesiologists physical status grade 2 or 3 and 60.14% underwent standard PD without vessel resection. Pancreatic adenocarcinoma was the most common pathologic type, with an incidence of 36.95%. The proportion of patients who underwent R0 resection for malignancy was 90.45%.

**Table 1:** Patient Demographics and Preoperative Variables (*n* = 301)

		Fluid balance quartile			
Characteristics	Total	1st	2nd	3rd	4th
Age (yr), mean ± SD	55.43 ± 10.90	54.85 ± 12.26	54.70 ± 11.38	56.79 ± 9.60	55.37 ± 10.26
Gender					
Female	155 (51.45)	37 (49.33)	41 (53.95)	35 (46.67)	42 (56.00)
Male	146 (48.55)	38 (50.67)	35 (46.05)	40 (53.33)	33 (44.00)
Weight (kg), mean ± SD	58.24 ± 10.10	58.61 ± 10.44	57.59 ± 10.33	58.15 ± 9.60	58.61 ± 10.18
BMI (kg/m^2^), mean ± SD	21.62 ± 3.07	21.65 ± 3.18	21.57 ± 2.94	21.54 ± 2.94	21.74 ± 3.25
Surface area (m^2^)	1.69 ± 0.19	1.70 ± 0.19	1.67 ± 0.20	1.69 ± 0.18	1.69 ± 0.18
ASA class					
1	9 (2.94)	2 (2.67)	4 (5.26)	2 (2.67)	1 (1.33)
2	121 (40.15)	32 (42.67)	31 (40.79)	30 (40.00)	28 (37.33)
3	171 (56.91)	41 (54.67)	41 (53.95)	43 (57.33)	46 (61.33)
Preoperative biliary drainage					
Yes	101 (33.54)	27 (36.00)	25 (32.89)	26 (34.67)	23 (30.67)
No	200 (66.46)	48 (64.00)	51 (67.11)	49 (65.33)	52 (69.33)
Surgical procedure					
Standard PD	181 (60.14)	49 (65.33)	46 (60.53)	45 (60.00)	41 (54.67)
Extended PD	68 (22.54)	16 (21.33)	17 (22.37)	15 (20.00)	20 (26.67)
PPPD	52 (17.25)	10 (13.33)	13 (17.11)	15 (20.00)	14 (18.67)
Vascular resection					
Yes	56 (18.50)	12 (16.00)	13 (17.11)	14 (18.67)	17 (22.67)
No	245 (81.50)	63 (84.00)	63 (82.89)	61 (81.33)	58 (77.33)
Pathological evidence					
Pancreatic adenocarcinoma	111 (36.95)	31 (41.33)	22 (28.95)	30 (40.00)	28 (37.33)
Pancreatic neuroendocrine tumor	10 (3.32)	4 (5.33)	2 (2.63)	3 (4.00)	1 (1.33)
IPMN	9 (2.99)	3 (4.00)	3 (3.95)	1 (1.33)	2 (2.67)
Ampullary adenocarcinoma	37 (12.29)	5 (6.67)	10 (13.16)	12 (16.00)	10 (13.33)
Ampullary adenoma	35 (11.63)	8 (10.67)	11 (14.47)	7 (9.33)	9 (12.00)
Duodenal adenocarcinoma	55 (18.27)	16 (21.33)	14 (18.42)	10 (13.33)	15 (20.00)
Cholangiocarcinoma	17 (5.65)	4 (5.33)	3 (3.95)	4 (5.33)	6 (8.00)
Mass-forming pancreatitis	19 (6.31)	3 (4.00)	8 (10.53)	6 (8.00)	2 (2.67)
Other	8 (2.66)	1 (1.33)	3 (3.95)	2 (2.67)	2 (2.67)
Tumor type					
Benign	81 (26.91)	19 (25.33)	27 (35.53)	19 (25.33)	16 (21.33)
Malignant	220 (73.09)	56 (74.67)	49 (64.47)	56 (74.67)	59 (78.67)
Grade (*n* = 220 malignancies)					
High	65 (29.54)	23 (30.67)	7 (9.21)	11 (14.67)	24 (32.00)
Moderate	112 (50.91)	24 (32.00)	31 (40.79)	31 (41.33)	26 (34.67)
Low	35 (15.91)	8 (10.67)	9 (11.84)	11 (14.67)	7 (9.33)
Not defined	8 (3.64)	1 (1.33)	2 (2.63)	3 (4.00)	2 (2.67)
Stage (*n* = 220 malignancies)					
0	0	0	0	0	0
IA	10 (4.55)	3 (4.00)	2 (2.63)	3 (4.00)	2 (2.63)
IB	16 (7.27)	5 (6.67)	4 (5.26)	2 (2.67)	5 (6.67)
IIA	32 (14.55)	9 (12.00)	6 (7.89)	9 (12.00)	8 (10.67)
IIB	117 (53.18)	29 (38.67)	27 (35.53)	29 (38.67)	32 (42.67)
III	22 (10.00)	4 (5.33)	5 (6.58)	7 (9.33)	6 (8.00)
IV	7 (3.18)	1 (1.33)	1 (1.32)	3 (4.00)	2 (2.67)
Not defined	16 (7.27)	5 (6.67)	4 (5.26)	3 (4.00)	4 (5.33)
Resection margin (*n* = 220 malignancies)					
R0	199 (90.45)	52 (92.86)	42(85.71)	51 (91.07)	54 (91.53)
R1	21 (9.55)	4 (7.14)	7 (14.2)	5 (8.93)	5 (8.47)

ASA, American Society of Anesthesiologists; BMI, body mass index; IPMN, intraductal papillary mucinous neoplasms. Data presented with Number (percentage) or Mean ± SD

The postoperative morbidity rate in the cohort was 56.48%, and the morbidities were predominantly Clavien–Dindo grades III and IV (Table 2). The most common complication was POPF, followed by DGE. The mortality rate was 3.65%. Fifteen patients required a reoperation.

**Table 2:** Postoperative outcomes

Outcome	
Morbidity	170 (56.48)
Mortality	11 (3.65)
Length of stay (d), mean ± SD	25.9 ± 10.9
ICU length of stay (d), mean ± SD	5.5 ± 2.9
Postoperative 0–24-h input (ml), mean (range)	3973 (1790–13,464)
Postoperative 24–48-h input (ml), mean (range)	4922 (2231–9804)
Postoperative 48–72-h input (ml), mean (range)	3383 (750–18,299)
Postoperative 72–96-h input (ml), mean (range)	4355 (750–14,585)
Postoperative 0–24-h output (ml), mean (range)	2400 (700–5600)
Postoperative 24–48-h output (ml), mean (range)	3369 (1095–7365)
Postoperative 48–72-h output (ml), mean (range)	3894 (985–7205)
Postoperative 72–96-h output (ml), mean (range)	4157 (476–7244)
Clavien Grade III	136 (45.18)
Clavien Grade IV	34 (11.30)
Surgical site infection	45 (14.95)
Pancreatic fistula	96 (31.89)
Grade A	71 (23.59)
Grade B/C	25 (8.31)
Bile leakage	4 (1.33)
Bowel leakage	5 (1.66)
Delayed gastric emptying	95 (31.56)
Grade A	63 (20.93)
Grade B	23 (7.64)
Grade C	9 (2.99)
SIRS	85 (28.24)
ARDS	34 (11.30)
Acute kidney injury	9 (2.99)
Hemorrhage	65 (21.59)
Heart failure/ Myocardial ischemia	23 (7.64)
Thrombosis	2 (0.66)
Hospital readmit	76 (25.25)
Reoperation	15 (4.98)

ARDS, acute respiratory distress syndrome; SIRS, systemic inflammatory response syndrome. Data presented with Number (percentage), Mean ± SD or Mean (range)

The fluid intake during the first 24 h after surgery ranged from 1790 to 13,464 mL, and the output ranged from 700 to 5600 mL. The fluid intake during the 24–48-h postoperative period ranged from 2231 to 9804 mL, and the output ranged from 1095 to 7365 mL. The fluid intake during the 48–72-h postoperative period ranged from 750 to 18,299 mL, and the output ranged from 985 to 7205 mL. Lastly, the fluid intake during the 72–96-h postoperative period ranged from 750 to 14,584 mL, and the output ranged from 476 to 7244 mL.

Univariate analysis revealed that patients in the higher quartiles of fluid balance were more likely to have higher morbidity rates and longer hospital and ICU LOS in the 0–24-, 24–48-, and 48–72-h postoperative periods than those in the lower quartiles; however, no significant differences were found in these parameters according to fluid balance in the 72–96-h postoperative period (Tables 3 and 4, Supplementary Tables 1 and 2). Additionally, compared with patients in the two lowest quartiles of fluid balance, those in the two highest quartiles had significantly higher mortality rates in the 24–48-h postoperative period, with trends toward increased incidences of POPF, DGE, SIRS, ARDS, hemorrhage, and heart failure and significant increases in these incidences in the 48–72-h postoperative period. After adjusting for the individual body surface area, the same trends were observed. Compared with patients in the first and second quartiles of fluid balance, those in the third and fourth quartiles had significantly higher incidences of POPF, DGE, SIRS, ARDS, hemorrhage, and heart failure in the 24–48- and 48–72-h postoperative periods.

**Table 3:** Surgical outcomes by 48-h fluid balance quartiles with surface area adjustment

	48-h fluid balance					48-h fluid balance (SA adjustment)				
	1st	2nd	3rd	4th	*p* value	1st	2nd	3rd	4th	p value
Morbidity	28	41	43	58	<0.001	28	42	44	56	<0.001
Mortality	0	1	5	5	0.051	0	1	3	7	0.014
Hospital LOS	23.8 ± 10.0	24.1 ± 11.2	26.5 ± 10.2	29.0 ± 11.8	0.012	23.6 ± 9.7	25.0 ± 11.8	26.3 ± 9.9	28.6 ± 11.9	0.036
ICU LOS	4.7 ± 2.0	4.9 ± 2.7	6.0 ± 3.4	6.4 ± 2.9	<0.001	4.7 ± 2.0	5.1 ± 2.8	5.6 ± 3.0	6.6 ± 3.3	<0.001
SSI	11	14	10	10	0.793	11	15	8	11	0.482
POPF	16	21	27	32	0.028	16	22	27	31	0.051
Bile leakage	0	3	1	0	0.112	0	3	0	1	0.112
Bowel leakage	2	1	1	1	0.892	2	1	1	1	0.892
DGE	13	18	28	36	<0.001	13	17	31	34	<0.001
SIRS	5	16	24	40	<0.001	5	16	25	39	<0.001
ARDS	2	7	10	15	0.008	2	7	10	15	0.008
AKI	1	1	5	2	0.174	1	1	3	4	0.372
Hemorrhage	8	10	14	33	<0.001	8	12	14	31	<0.001
HF/MI	0	1	7	15	<0.001	0	2	5	16	<0.001
Liver failure	1	2	8	6	0.041	1	4	4	8	0.102
Thrombosis	0	1	1	0	0.572	0	1	1	0	0.572
Readmission	14	19	15	28	0.035	13	19	16	28	0.031
Reoperation	1	2	3	9	0.012	1	2	4	8	0.040

AKI, acute kidney injury; ARDS, acute respiratory distress syndrome; DGE, delayed gastric emptying; HF, heart failure; ICU, intensive care unit; LOS, length of stay; MI, myocardial infarction; POPF, postoperative pancreatic fistula; SA, surface area; SIRS, systemic inflammatory response syndrome; SSI, surgical site infection

**Table 4:** Surgical outcomes by 72-h fluid balance quartiles with surface area adjustment

	72-h fluid balance					72-h fluid balance (SA adjustment)				
	1st	2nd	3rd	4th	p value	1st	2nd	3rd	4th	p value
Morbidity	29	37	47	57	<0.001	29	40	44	57	<0.001
Mortality	0	1	2	8	0.002	1	0	1	9	<0.001
Hospital LOS	22.3 ± 9.3	26.8 ± 9.3	26.9 ± 12.8	27.3 ± 11.6	0.014	22.4 ± 9.3	26.5 ± 9.2	27.1 ± 12.9	27.4 ± 11.5	0.015
ICU LOS	4.4 ± 2.4	5.0 ± 2.1	5.5 ± 2.8	7.0 ± 3.4	<0.001	4.5 ± 2.5	5.2 ± 2.1	5.2 ± 2.3	7.2 ± 2.9	<0.001
SSI	8	10	18	9	0.085	8	10	17	10	0.176
POPF	17	18	25	36	0.002	17	19	23	37	0.002
Bile leakage	2	0	1	1	0.563	2	0	1	1	0.563
Bowel leakage	1	2	2	0	0.528	1	2	2	0	0.528
DGE	16	20	24	35	0.006	15	21	23	36	0.002
SIRS	8	17	20	40	<0.001	8	17	19	41	<0.001
ARDS	2	4	9	19	<0.001	2	5	7	20	<0.001
AKI	0	1	1	7	0.003	0	2	0	7	0.002
Hemorrhage	7	13	16	29	<0.001	7	13	15	30	<0.001
HF/MI	1	1	2	19	<0.001	1	1	1	20	<0.001
Liver failure	2	1	4	10	0.007	2	1	3	11	0.001
Thrombosis	1	0	1	0	0.567	1	0	1	0	0.567
Readmission	12	17	20	27	0.038	12	17	20	27	0.038
Reoperation	1	1	5	8	0.020	1	1	5	8	0.020

AKI, acute kidney injury; ARDS, acute respiratory distress syndrome; DGE, delayed gastric emptying; HF, heart failure; ICU, intensive care unit; LOS, length of stay; MI, myocardial infarction; POPF, postoperative pancreatic fistula; SA, surface area; SIRS, systemic inflammatory response syndrome; SSI, surgical site infection

**Fig. 1:**
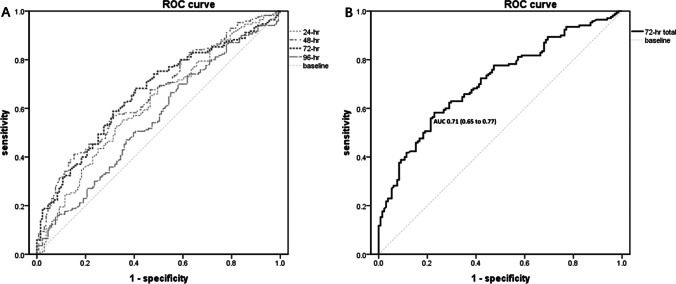
Receiver operating characteristic (ROC) curves of fluid balance for the prediction of morbidities. (A) Fluid balance ROC curve for each period (postoperative 24, 48, 72, and 96 h). (B) Fluid balance ROC curve for the total postoperative 72-h interval

Receiver operating characteristic curves were created to evaluate the relationship between fluid balance and morbidity at each postoperative time interval. The areas under the curve for the 0–24-, 24–48-, 48–72-, and 72–96-h postoperative periods were 0.62, 0.66, 0.67, and 0.56, respectively (*P* = 0.001, *P* < 0.001, P < 0.001, and *P* = 0.095, respectively). Sensitivity and specificity testing for each period was performed to determine the best cutoff point for discriminating between excessive and non-excessive fluid balance. The optimal fluid balance values were 1730 mL for the 0–24-h postoperative period (sensitivity, 55.30%; specificity, 64.89%), 1684 mL for the 24–48-h postoperative period (sensitivity, 56.47%; specificity, 69.47%), and 616 mL for the 48–72-h postoperative period (sensitivity, 68.24%; specificity, 59.54%) (Fig. 1A). When the overall fluid balance in the 0–72-h postoperative period was considered, the area under the curve was 0.71 (0.65–0.77) and the optimal fluid balance value that discriminated hospital morbidity was 4212 mL (sensitivity, 58.24%; specificity, 77.10%) (Fig. 1B). Patients with an excessive overall fluid balance in the 0–72-h postoperative period had a higher hospital morbidity rate than those with a non-excessive fluid balance (76.7% vs. 41.3%, *P* < 0.001).

## Discussion

The negative effects of excessive fluid overload in the postoperative period have long been recognized [13–16]. A contemporary randomized study investigating fluid regimens in patients undergoing colorectal surgery found that a restrictive fluid strategy not only led to decreased cardiopulmonary morbidity but also reduced the incidence of tissue-healing complications [17]. Similarly, a recent prospective cohort study that focused on major surgeries found that patients with an excessive intraoperative fluid balance had higher hospital mortality rates than those with a non-excessive intraoperative fluid balance [18].

The present study examined the role of postoperative fluid balance in patients undergoing PD, with a focus on four consecutive postoperative time intervals. Compared with patients in the lower quartiles of fluid balance, those in the higher quartiles had increased incidences of POPF, hemorrhage, DGE, ARDS, SIRS, and overall morbidity, in addition to longer ICU and hospital LOS. The differences in the incidences of these adverse events according to fluid balance quartiles were more pronounced in the 24–48- and 48–72-h postoperative periods. The same conclusions were obtained after adjusting for the individual body surface area.

Various studies have discussed how to best regulate and manage fluid balance during the perioperative period, and the recommendations have changed from the initial regimen of liberal fluid intake to the currently used restricted fluid balance regimen [19]. Many studies have focused on whether perioperative fluid balance management or maintenance of a positive or negative fluid balance in the early postoperative period positively or negatively affects the morbidity and mortality rates in abdominal surgery [20–22]. The present results demonstrated a morbidity rate of 56.48% in the entire cohort of patients who underwent PD, with a rate of 76.00% in the highest quartile of fluid balance in the 0–72-h postoperative period compared with 38.67% in the lowest quartile. Additional analyses about the timing of morbidities indicated that patients with early postoperative complications received more fluid. Only 18% of morbidities (31 of 170) were suspected or diagnosed within the first 72 h after surgery. This suggests that a higher fluid balance, rather than a larger fluid volume received, was associated with a higher incidence of morbidities.. In other words, failure to mobilize fluid may be an early indicator of impending complications.

POPF is a major concern after PD. In our study, the incidence of POPF was higher in patients in the higher quartiles of fluid balance than in those in the lower quartiles throughout the entire 0–72-h postoperative period. This trend was particularly obvious in the 48–72-h period, during which nearly half of the patients (36 of 75) in the highest quartile of fluid balance had POPF compared to less than a quarter of patients in the lowest quartile (17 of 75). It seemed that a higher fluid balance after PD was associated with a higher POPF rate, which was consistent with the findings reported by Wang et al. [23]

In 1972, an animal study showed that increasing the fluid balance aggravates tissue edema, which impairs oxygen diffusion, decreases tissue oxygen tension, and leads to worse healing [24]. Another study reported that the quantity of infusion significantly affects the functional and structural stability of intestinal anastomoses in the early postoperative period, particularly from postoperative days 3 to 5 [25]. As the stability and quality of intestinal anastomosis influence the insufficiency rate, volume overload may have deleterious effects on anastomotic healing and postoperative complications in digestive surgery because of marked bowel wall edema. Thus, restricting fluid balance may decrease the degree of bowel edema, which would benefit anastomotic healing to some extent.

Excessive fluid intake is associated with cardiopulmonary events after a major surgery. A perioperative positive fluid balance of >2000 mL has been reported to increase the risk of cardiovascular complications by 2.5 times [26]. In the current study, patients in the highest quartile of fluid balance had a much higher incidence of heart failure or myocardial infarction in the 24–48- and 48–72-h postoperative periods than those in the lower quartiles. Although fluid administration can increase cardiac output, an excessive amount of fluid can subsequently depress ventricular function and increase cardiac morbidity [27]. Furthermore, surgical trauma increases the permeability of the capillaries, leading to a large amount of exudation. Our data revealed that 11.30% of the patients who underwent PD developed ARDS postoperatively. Unsurprisingly, patients in the higher quartiles of fluid balance were more likely to develop ARDS in the 0–24-, 24–48-, and 48–72-h postoperative periods than those in the lower quartiles. A previous study reported that the adverse effects of volume overload are more evident in the lungs, where fluid resuscitation can lead to acute pulmonary edema, compromising gas exchange and increasing the patient’s susceptibility to infection. Additionally, pulmonary function may be impaired by the accumulation of interstitial fluid, which can contribute to the development of pulmonary edema, atelectasis, pneumonia, or even respiratory failure.^28^

The present study had some limitations. First, this was a single-center retrospective analysis; thus, the inherent bias in this type of research should be considered. Second, daily weight changes were not thoroughly investigated, although such data can also provide evidence of the degree of fluid overload and can be used to scrutinize the results. Third, the effects of fluid type (crystalloid, colloid, or blood products) and quantitative data on fluid intake were not evaluated.

## Conclusion

The present study investigated a large cohort of patients who underwent PD and found that an increased fluid balance in the early postoperative period (0–72 h after surgery) was associated with increased incidences of overall morbidity, POPF, DGE, and ARDS, as well as longer hospital and ICU LOS. In patients with a high postoperative fluid balance, particularly in the first few days, surgeons should pay close attention to the occurrence of complications.

## Supplementary Information

ESM 1 (DOCX 64 kb)
